# For which clinical rules do doctors want decision support, and why? A survey of Dutch general practitioners

**DOI:** 10.1177/1460458217740407

**Published:** 2017-11-17

**Authors:** Stephanie Medlock, Saeid Eslami, Marjan Askari, Derk L Arts, Esther M van de Glind, Henk J Brouwer, Henk C van Weert, Sophia E de Rooij, Ameen Abu-Hanna

**Affiliations:** Amsterdam Public Health Research Institute, The Netherlands; Amsterdam Public Health Research Institute, The Netherlands; Mashhad University of Medical Sciences, The Islamic Republic of Iran; Amsterdam Public Health Research Institute, The Netherlands; Universiteit Utrecht, The Netherlands; Amsterdam Public Health Research Institute, The Netherlands; University of Amsterdam, The Netherlands; Amsterdam Public Health Research Institute, The Netherlands; University of Amsterdam, The Netherlands; University of Amsterdam, The Netherlands; University of Groningen, The Netherlands; Amsterdam Public Health Research Institute, The Netherlands

**Keywords:** clinical, decision support systems, general practitioners, medication alert systems, surveys

## Abstract

Despite the promise of decision support for improving care, alerts are often overridden or ignored. We evaluated Dutch general practitioners’ intention to accept decision support in a proposed implementation based on clinical rules regarding care for elderly patients, and their reasons for wanting or not wanting support. We developed a survey based on literature and structured interviews and distributed it to all doctors who would receive support in the proposed implementation (n = 43), of which 65 percent responded. The survey consisted of six questions for each of 20 clinical rules. Despite concerns about interruption, doctors tended to choose more interruptive forms of support. Doctors wanted support when they felt the rule represented minimal care, perceived a need to improve care, and felt responsible for the action and that they might forget to perform the action; doctors declined support due to feeling that it was unnecessary and due to concerns about interruption.

## Background

Computerized clinical decision support is any computerized tool designed to help clinicians make clinical decisions.^1^ Decision support often takes the form of a computer-generated alert in response to a user’s action, for example, a warning which is displayed if the user prescribes a medication to a patient with a known allergy to that medication. It is one of the most promising strategies for improving patient care^2^ and the ability to provide such support is viewed as one of the important benefits of using a computerized patient record system.^3^ However, this benefit is often not fully realized in practice. Alerts are often overridden, in the Netherlands^4^ and elsewhere.^5–7^

There are many reasons why a clinician might override an alert. Clinicians may generally dislike guidelines and clinical rules, due to a sense of loss of autonomy^8–10^ or concern that their use will lead to “cookbook medicine.”^8,9^ Previous experience with dysfunctional decision support systems can contribute to poor acceptance of new alerts.^10^ Also, the presence of too many alerts can lead to ignoring all alerts (“alert fatigue”),^4,11^ especially too many false-positive alerts.^12^ The use of less-interruptive styles of decision support for some alerts has been proposed to help reduce alert fatigue,^13^ which may be particularly important in light of the possible adverse effects of interruption on the quality of care.^14^

Dutch general practitioners (GPs) were among the first to widely adopt electronic patient records,^15^ and today, some form of clinical decision support is used in 89 percent of Dutch general practices.^16^ The HAG-net-AMC (General Practice-network Academic Medical Center), a regional network of GPs participating in research and education, is planning to implement decision support based on a set of 81 “if-then” clinical rules pertaining to geriatric medicine. The rules were selected using a Delphi method for their relevance to Dutch general practice and are in the form of “condition-action” rules (e.g. “If a (vulnerable) elder is prescribed an ACE inhibitor” (condition), “then s/he should have serum creatinine and potassium monitored within 2 weeks after initiation of therapy and at least yearly thereafter” (action)).^17^ The clinical rules serve as concrete examples of clinical tasks proposed for decision support.

We developed and administered a survey to learn which of these rules doctors want supported, and the reasons behind their choices. The aim is to gain insight into the motivation for prospectively accepting or declining support, as well as gain information to assist in designing support that better suits the doctors’ workflow and expectations.

## Materials and methods

**Figure 1.**
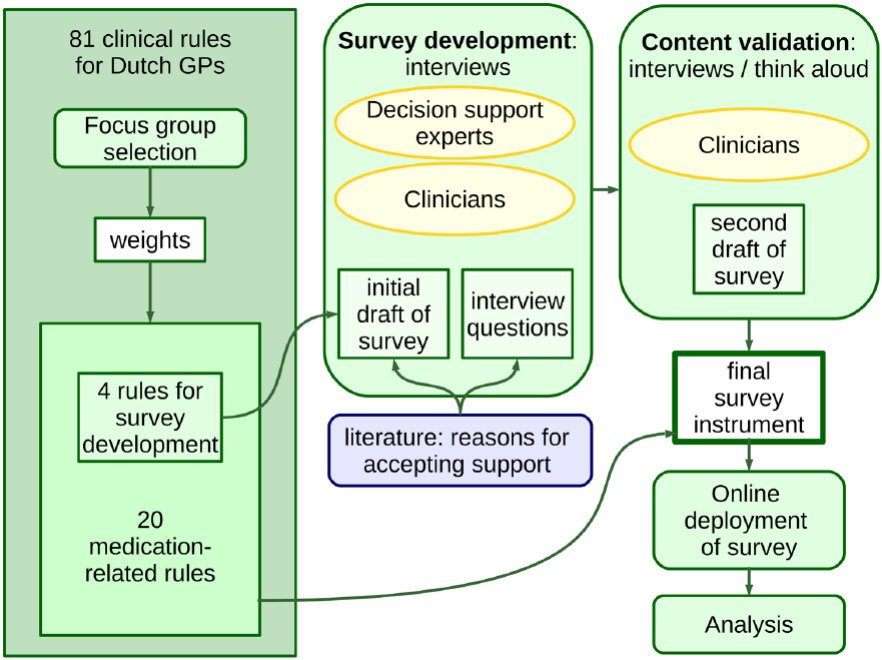
Survey development and deployment process. The survey was developed based on input from the literature and interviews with clinicians and experts in decision support. The content was validated by conducting a second set of interviews, including a concurrent “think-aloud” process. The final survey was deployed online.

A summary of the survey development and deployment process is presented in Figure 1.

### Selection of clinical rules for the survey

The complete set of 81 rules was prioritized for the decision support implementation by a focus group of eight doctors from the practice group. This process is described in detail elsewhere,^18^ but briefly, participants independently indicated whether they would turn support “on” or “off” for each rule and then rated the importance of the rule on a scale from 1 to 10. The decision was made to focus on medication-related rules; 23 rules mentioned medications or specific medications. Three rules were eliminated because they were conceptually similar, leaving 20 rules to include in the survey. This selection was checked to ensure that it included rules which had been rated as both high and low importance by the focus group, so as to capture both reasons for wanting and not wanting support in the survey.

### Survey development

We expected that general attitudes and previous experience with decision support could globally influence responses to individual rules; therefore, we also administered an “Attitudes and Experience” questionnaire, the details of which have been published previously.^19^

The survey was developed by conducting structured interviews with two experts in decision support systems, a hospital specialist, a hospital resident, and two GPs. We included experts in decision support and hospital physicians with the expectation that they had had experience with different systems than the GPs and thus could contribute a broader perspective on reasons why systems are ultimately accepted or rejected. The interviews were structured in three parts, described in detail in Table 1.

**Table 1.** Interview structure and questions.

Part 1: attitudes, experiences, and expectations
“What experience do you have with clinical decision support?”
“What do you think about clinical decision support in general?”
“What do you expect from clinical decision support in the next year or two?”
“What are the advantages of clinical decision support?”
“What are the disadvantages?”
Part 2: open questions about each of four example clinical rules
“Would you want regular feedback or decision support based on this rule?,”
“Why or why not?”
“What factors do you think about when considering your decision?”
Part 3: draft survey using same four example rules (based on literature^8,10,20^ and input from an expert in medical psychology)

The four rules used in the interviews were selected to be representative of rules relating to medication management.^21^ We did not record the interviews, because our clinical advisors felt this would likely lead to social desirability bias. Interviewees were asked to write down their answers and the interviewer noted any verbal comments on the same paper. All interviews were conducted by one researcher (SM), with the first three interviews observed by a second researcher (SE). Results from the interviews were discussed in meetings with four medical informaticians (SM, SE, MA, and AA) and used to identify new reasons not found in the literature and eliminate reasons which interviewees said were not relevant. Comments reflecting general attitudes or experience were added to the “Attitudes and Experience” questionnaire.^19^

Content validity of the resulting survey was assessed by conducting a second set of interviews. We used a concurrent think-aloud verbal protocol:^22^ doctors who had not participated in the previous interviews were asked to “think aloud” while filling in the survey, using the four example rules. All interviews were conducted by one researcher and were not recorded. The researcher noted their comments on note paper that was visible to the interviewee. The researcher did not prompt the subject while they were filling in each section, except to remind them to “think aloud.” At the end of each section, subjects were also asked specifically if they would like to add or change anything. Any changes suggested would then be incorporated into the survey for use in the next interview. If any part of the survey was unclear or if new reasons were suggested, this part of the survey would be revised. The process would end after three consecutive interviews with no changes to the survey. The final formulation of the survey was reviewed by two experts in medical informatics (SE and AA) and two clinicians (SdR and EvdG) prior to deployment.

### Deploying the survey

The survey was translated to the Dutch language by a native Dutch speaker and resident in geriatric medicine (EvdG) and back-translated by a native English speaker (SM). It was deployed as an anonymous, online survey among all GPs participating in the HAG-net-AMC and thus to all potential future users of the system. Participants were invited by an email from the secretary of the steering committee of the HAG-net-AMC on 6 July 2011 and reminded with two follow-up emails 2 and 4 weeks later. Responses were collected up to 3 months after the initial email. To preserve anonymity, we did not attempt to identify participants. To eliminate potential bias due to the order of presentation of the rules, the order was randomized for each participant.

### Analysis

All statistical analyses were performed using R 3.0.1.^23^ The difference in medians of ordinal variables was assessed with the Mann–Whitney U test and difference in proportions with Pearson’s chi-square test or Fisher’s exact test. Multivariate analysis was performed using logistic regression models. Adjusted odds ratios (aORs) are adjusted for age and gender, unless otherwise noted. Generalized estimating equations (GEEs) with logistic regression were used to account for the effect of clustering by user, with results reported as the odds ratio (OR).^24^ The binomial test was used to determine whether the choice of reasons for wanting or not wanting support differed significantly from chance. Free-text comments were assessed separately by two researchers (SM and SE), with disagreements resolved by a third researcher (AA).

## Results

### Survey development: interviews

Table 2 shows the reasons for wanting or not wanting support that were suggested in the interviews.

**Table 2.** Reasons for wanting or not wanting support suggested by the interviews, with selected quotations.

Concepts		Quotations
Reasons for wanting support	Help recognize that care is needed	“Otherwise you don’t know if you missed something”
	Avoid forgetting Prevents harm to the patient	“That I won’t forget … that I won’t make a terrible mistake.” “It could harm the patient [if it’s forgotten]”
	Action is needed quickly	“Timely response”
	Helps adherence to (required) quality indicators	“We have quality indicators that are obligatory for IGZ but are not followed.”
Reasons for not wanting support	Feel that support is needed for others but not for themselves Will recognize when care is needed Would not forget	“This is a good rule … I know this, so I don’t need support, but on the wards … there’s always a chance that it could happen.” “My colleagues include nurses, trainees … I want support for some but not all of my colleagues” “I don’t need it because I don’t forget it … this does happen in my practice though.”
	Interruption, Wrong advice	“It can hold you up in the work” “Disturb work … lead to the wrong decisions … lead to bad outcomes” “What is the expected frequency of these alerts? I don’t want to be annoyed by the alerts … What is the availability and reliability of the data?”
	Someone else is responsible for the action	“The QI may not apply to me, I may not be the decision-maker” “The POH does this”

### Survey development: survey instrument

The resulting survey consisted of six questions for each clinical rule, with the phrasing of the question modified to reflect the conditions and actions of each rule. The first three questions establish whether the respondent agrees in principle with the need to improve performance: “This rule constitutes minimal care that every general practitioner should know,” “This rule *should be* followed for ___% of my older patients where [the condition applies],” and “When this rule should be followed, I think it is actually followed in ___% of the cases in my practice,” where respondents can give an estimate in 10 percent intervals. The fourth question addresses alert design: the respondent is asked to choose between two or three options (e.g. a pop-up vs an item added to a to-do list), with at least one less-interruptive option offered for each rule. Finally, the respondent was asked to decide whether they would turn support for this rule “on or off,” and choose from a list of reasons why they made this decision. Space was also provided to fill in comments or additional reasons. An example of the questions for one clinical rule is given in Appendix 1, with annotations indicating the main source of the question (literature source or interviews). Both draft and final versions of the instrument are available from the authors upon request.

### Results from survey of GPs

Demographic characteristics of respondents are reported in Table 3. In total, 43 GPs were invited to complete the survey, of which 28 completed all questions for at least one clinical rule (65%), and 20 completed the survey (47%) for all clinical rules, and the remainder completed questions for 1–6 rules (median 2). The demographics of respondents were representative of the study population. Subjects who completed the survey tended to be older than those who finished only part of the survey (median age categories 50–60 years vs 40–50 years, 95% confidence interval (CI) = −20 to 0, p = 0.02).

**Table 3.** Demographics of participants and population.

	Invited	Responded
n	43	28
Gender	60% female	60% female
Age	Mean 50 years	Median 50–60 years

#### Choice of clinical rules to support

In response to the question, “On my computer, I would turn support on/off,” more than 50 percent of respondents indicated that they would turn support “on” for 17/20 rules. Responses per rule ranged from 28 to 88 percent in favor of support. Responses were not significantly different between those who did and did not complete the survey (64% positive for those who completed the survey vs 76% for those who did not (aOR = 0.90, CI = 0.67−1.20, p = 0.47)). The proportion of rules selected to be supported was not significantly associated with responses to the Attitudes and Experiences questionnaire^19^ (aOR = 1.02, CI = 0.99−1.05, p = 0.17).

#### Type of support

For each clinical rule, at least two different types of support were suggested, including at least one option for a more interruptive form of support (e.g. a pop-up) and one for a less-interruptive form of support (e.g. adding the item to a task list). More than one answer was allowed, and respondents were allowed to choose a type of support whether or not they wanted support for the rule. Results are summarized in Table 4.

**Table 4.** Type of support selected.

	All rules	Maximum (per rule)	Minimum (per rule)
Interruptive	54%	75%	7%
Noninterruptive	35%	80%	10%
Both	12%	27%	5%

More-interruptive forms of support were chosen significantly more often than less-interruptive forms of support (χ^2^ = 23, p < 0.0001). However, this also varied significantly between rules (χ^2^ = 43, p = 0.001). Noninterruptive options were preferred for four rules. A type of support was chosen in 95 percent of responses where support was wanted and 58 percent of responses where support was declined. A noninterruptive option was selected in 51 percent of answers when support was wanted and 40 percent when support was not wanted, though this difference was not significant (OR = 0.638, CI = 0.35–1.16, p = 0.14, clustered by user).

#### Establishing the need to improve performance

The need to improve performance was assessed through three questions, summarized in Table 5.

**Table 5.** Perceived need to improve performance.

	All rules	Maximum (per rule)	Minimum (per rule)	Range (all responses)
This rule constitutes *minimal care*	Agree 90%, disagree 10%	Agree 100%, disagree 0%	Agree 66%, disagree 44%	N/A
This rule *should be followed* for __% of patients …	100% (13/20 rules)	100%	80%	0% to 100%
This rule *is followed* in practice in __% of patients …	70%	90%	40%	0% to 100%
Perceived gap in compliance (% *should be followed *− % *is followed*)	20%	30%	0%	−70% to 80%^a^

a There was no gap for 25 percent of responses, and a negative gap for 1 percent of responses (n = 5).

**Figure 2.**
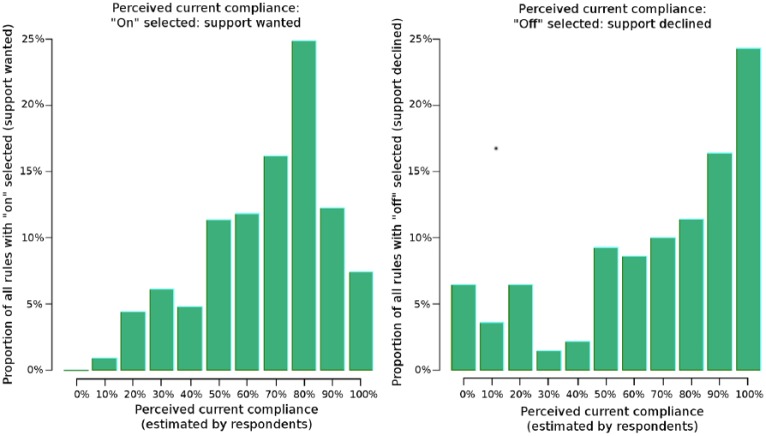
Histogram of responses to the question, “This rule is followed in practice in __% of cases” for rules where support was wanted (left, n = 229) and declined (right, n = 140). The x-axis represents the response categories (0%, 10%, 20%, …, 100%) and the y-axis represents the percentage of responses in that category. Respondents tended to want support for rules when they estimated current compliance to be in the high-middle range and decline support when they estimated current compliance to be low or very high.

Feeling that the rule constitutes *minimal care* was positively associated with wanting support (OR = 2.68, CI = 1.78−4.04, p < 0.0001, clustered by user), as was feeling that the rule *should be followed* (OR = 10.3, CI = 3.53−29.9, p < 0.0001, clustered by user). Feeling that the rule *is followed* (perceived current compliance) had a nonlinear relationship with choosing the rule for support (Figure 2). There was no significant linear relationship (aOR = 0.95, CI = 0.41−2.18, p = 0.91). The difference between how often the rule is followed and how often it should be followed (*perceived gap in compliance*) was also very strongly associated with wanting support (OR = 28.8, CI = 3.46−239.0, p = 0.002, clustered by user). None of these factors were associated with the selection of interruptive versus noninterruptive support.

#### Reasons for wanting support

**Figure 3.**
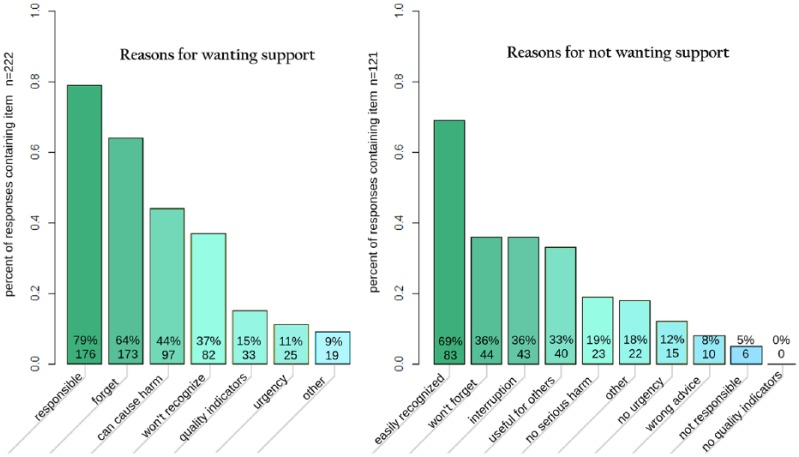
Frequency of selection of reasons for wanting or declining support. Respondents could choose any number of reasons, no reasons, or fill in a free-text field (“other”). Percentages are the percentage of responses which included this item. The full text of the reasons is given in Tables 3 and 4.

Of the rules where respondents indicated that they wanted support, respondents gave a median of two reasons, and 93 percent of responses included at least one reason (Figure 3). Responses that were chosen significantly more often were a sense of responsibility, concern about forgetting to perform the action, and belief that failure to perform the action will harm the patient (Table 6). Selection of all responses differed significantly from chance (p < 0.01).

**Table 6.** Reasons for wanting support.

Answer	n (%, 95% CI)
I am responsible for this action	176/238 (73, 67%–79%)
I might forget to perform this action	143/238 (60, 53%–66%)
Failure to perform this action can lead to harm for the patient	97/238 (41, 34%–47%)
Immediately	17/238 (7)
After days	28/238 (12)
After weeks	45/238 (19)
After months	31/238 (13)
I often do not realize when a patient meets these conditions and needs this care	82/238 (34, 28%–40%)
This rule is relevant for performance indicators	33/238 (14, 10%–19%)
When this rule applies, rapid action is needed	25/238 (11, 7%–15%)
Free-text comments	176/238 (73, 67%–79%)

CI: confidence interval.

n = total number of times this response was chosen; the percentage is the percentage of times this answer was chosen out of the times it was displayed, regardless of whether any reasons were given.

#### Reasons for declining support

Of the rules where respondents indicated that they did not want support, respondents gave a median of two reasons, and 95 percent of responses included at least one reason (Figure 3). Responses that were chosen significantly more often were: feeling that they would know when the action is needed, not forget to perform the action, concerns about interruption, and feeling that support could be useful for others rather than themselves (Table 7). Selection of all responses differed significantly from chance (p < 0.01).

**Table 7.** Reasons for declining support.

Answer	n (%, 95% CI)
I can easily recognize when this treatment is needed	83/121 (60, 50%–67%)
I will not forget to perform this action	44/121 (31, 24%–40%)
Decision support will interrupt my way of working	43/121 (30, 23%–39%)
I don’t want support, but it could be useful for others	40/121 (28, 21%–37%)
Failure to follow this rule will not result in unacceptable harm to the patient	23/121 (16, 11%–24%)
No rapid action is needed (I can do it later)	15/121 (11, 6%–16%)
Decision support is frequently wrong	10/121 (7, 3%–13%)
I am not responsible for this action	6/121 (4, 2%–9%)
There are no performance indicators relevant to this rule	0/121 (0, 0%–3%)
Free-text comments	22/121

CI: confidence interval.

n = total number of times this response was chosen; the percentage is the percentage of times this answer was chosen out of the times it was displayed, regardless of whether any reasons were given.

The two reviewers disagreed as to whether only 3 of the 42 free-text comments constituted a new reason, which were then reviewed by the third reviewer. After discussion, one comment about wanting support was interpreted as constituting a new reason, namely, “I will answer yes if it is accepted policy of the NHG.” The NHG (College of General Practitioners) produces the national guidelines for general practice; thus, this reason can be generalized as “It supports our accepted guidelines.” None of the comments about declining support were interpreted as new reasons.

## Discussion

A survey was developed consisting of six questions about each of 20 clinical rules, covering whether the respondent agrees in principle with the need to improve performance on this clinical rule, alert design choices, and whether the respondent would personally want support for the rule and their reasons for that choice. Responses were received from 65 percent of the 43 practitioners invited, and 47 percent completed the survey. Despite expressing concerns about interruption, the doctors chose an interruptive style of support (e.g. pop-ups) in 54 percent of responses, compared to 35 percent for less-interruptive options. Feeling that the rule represents minimal care was associated with wanting support, as was feeling that the rule should be followed in a higher percentage of cases and a larger perceived gap between how often the rule is followed and how often it should be followed in practice. The most common reasons given for wanting support were a sense of responsibility, concern about forgetting to perform the action, and belief that failure to perform the action will lead to harm for the patient. One additional reason was suggested in the comments, namely, that the support facilitates following accepted guidelines. The most common reasons for declining support were feeling that they would already know when action is needed, not forget to perform the action, concerns about interruption, and feeling that support could be useful for others rather than themselves.

To our knowledge, this study represents the first structured, prospective investigation of clinicians’ intention to accept decision support for specific clinical rules and the reasons for those choices. An important contribution of this study is the introduction of the survey instrument. We used both literature resources and interviews in the development of this survey to ensure content validity. To reassure our interview subjects of their anonymity, we chose not to record the interviews. However, this could have introduced bias, since only one researcher conducted a majority of the interviews. This was mitigated by having the note paper always visible to the interview subject. We also chose to focus only on medication-related rules for this survey. Medication data quality is generally sufficient to build decision support; thus, it was likely that these rules could be implemented, and the authors felt that it might be disappointing to the participants if many of the rules selected in the survey were later discovered to be unimplementable. With 20 rules, the survey took about 30 min to complete, and the authors felt that adding more rules would reduce response rate. However, results could differ for other clinical domains, and this should be investigated in future studies.

The primary limitation is the small sample of doctors surveyed. However, we chose to limit the survey to doctors in the practices where the decision support intervention is planned, because this makes the survey highly relevant for this population. This may have also contributed to our high response rate. However, it is possible that other results would be obtained from different groups of GPs or from other types of clinicians such as hospital specialists. Further study is needed to determine whether these findings are also seen in other groups of clinicians. It is possible that responses were affected by social desirability bias, although we tried to minimize this by ensuring the anonymity of all participants. However, this meant that we were unable to detect whether the eight doctors who participated in the focus group also filled in the online survey. Given that doctors probably filled in the online survey *or* participated in the focus group, and not both, it is likely that our response rate is even higher (80% rather than 65%). Social desirability bias was also mitigated by framing the question as a personal decision (“Would you turn support on or off?”) rather than something that affects the whole practice (e.g. “Should decision support be implemented for this rule?”). We chose to frame it as a yes/no question rather than asking participants to rank or rate the rules by importance because of the cognitive difficulty inherent in ranking many items.^25^ Finally, there is a possibility of participation bias, although the survey was presented as a platform for voicing either support or objections to the new system.

A recently published study describes a process for the related task of prioritizing implementation of medication-related decision support alerts.^26^ The authors identified six factors to determine whether an alert should be implemented: quality of care, legal and regulatory compliance, organizational liability, daily workload, patient safety, and likelihood of occurrence. In terms of these constructs, patient safety was the primary determining factor in our subjects’ intention to accept support, followed by quality of care and workload concerns. This likely partly reflects the difference in objectives (alert prioritization vs intention to accept support) and partly differences in the health-care systems (the United States and the Netherlands).

Other studies have retrospectively evaluated the reasons for alert override and acceptance.^7,27^ Zheng et al.^27^ identified six constructs affecting acceptance: performance expectancy (whether the alerts are expected to help), ease of use, effort expectancy (to perform the recommended action), social influence, facilitating conditions, and perceived use of the alerts. Seidling et al.^7^ conducted a retrospective analysis of drug–drug interaction alerts and found that knowledge quality, display characteristics, text, setting, patient age, dosing alerts, alert frequency, and severity level all impacted acceptance. A common theme among these works and our study is that clinicians conceptually accept support if they anticipate that the alerts will help them to provide better care.

There seemed to be a nonlinear relationship between wanting support and responses to the question, “This rule *is* followed for ___% of patients.” Support was wanted for rules with a perceived current compliance in the high-middle range, and support was not wanted for rules with either a low or very high perceived current compliance (see Figure 2). It may be that low perceived compliance implies that the doctor does not agree with the rule, and high perceived compliance implies that the doctor believes he can comply with the rule without needing support.

Selection of the reason “This rule is (not) relevant for performance indicators” was markedly lower than the other reasons, even though several interviewees felt it was important. This may be because at the time of our interviews coincided with the introduction of new national performance indicators. We considered the free-text comment “It supports our accepted guidelines” to be different because our subjects likely think of “performance indicators” as external measures, whereas guidelines are used internally. Further research is needed to determine an optimal set of questions with utility for determining when decision support is likely to work and particularly whether a survey such as this one can help determine what kind of decision support will be most effective.

Although we encouraged our participants to “think outside the pop-up box” by describing both an interruptive alert and an alternative, most participants chose more interruptive alerts. The type of support chosen did not appear to be correlated with wanting support or any of the other factors measured in our survey. However, there could be other unmeasured attributes of our rules influencing this choice. Alternatively, this choice may be due to awareness of a higher response rate for alerts which require immediate attention^7,28^ or thinking about the alert in isolation or simply greater familiarity with this form of support. We know that too many alerts will lead to alert fatigue, but it is not currently known how many is “too many,” and what circumstances might mitigate this effect. The results of this survey suggest that simply having clinicians select the type of support from a multiple-choice list will not by itself solve the issues of alert overload and alert fatigue; we must design alerts with an understanding of the clinical workflow, the needs of daily practice, and the capabilities of computers and offer solutions that integrate with the care process.^29^ Further research is needed in determining which rules justify interruptive forms of support and, for the others, what kind of support can be offered that is both less interruptive and effective.

## Conclusion

A new survey instrument is introduced to prospectively assess clinicians’ intention to accept proposed decision support. The instrument includes measures of the perceived agreement with the rule and need to improve compliance, proposed designs for support, and whether the respondent personally wants support and their reason for that choice. The instrument was piloted with 20 clinical rules proposed for inclusion in a decision support system, in a population of 43 GPs. Feeling that the rule represents minimal required care, that the rule should be followed in a larger percentage of patients, and a perceived gap between how often the rule is currently followed and how often it should be followed were all associated with wanting support. The reasons given for wanting support included a sense of responsibility, concerns about forgetting to perform the action, and belief that failure to perform the action will lead to harm for the patient. Reasons given for declining support included feeling that they would know when to act and would not forget to perform the action, concerns about interruption, and feeling that support could be useful for others. Despite the concerns about interruption, doctors tended to choose pop-ups over other forms of support, implying that asking clinicians to choose the type of support is not sufficient to address issues of alert overload and alert fatigue.

## Appendix 1

### Example question for a clinical rule

                             part 3/3, page 2/20

               example

***IF*** *a (vulnerable) elder is prescribed an NSAID*, ***THEN*** *the GP record should indicate whether or not s/he has a history of 1) gastrointestinal bleeding or ulcers and 2) renal insufficiency or 3) heart failure AND, if a history is present, the general practitioner should document justification of NSAID use.*

**Table:**

This rule should be followed for:^8^	(dropdown) 100% (all), 90, 80, 70, 60, 50, 40, 30, 20, 10, 0% (none)	of my older patients that start therapy with an NSAID
When this rule should be followed, I think it is actually followed in [i]	(dropdown) n/a.,100% (all), 90, 80, 70, 60, 50, 40, 30, 20, 10, 0% (none)	of the cases in my practice
This rule constitutes minimal care for elderly patients that every general practitioner should know.^8^ □ no □ yes		

What sort of support would you prefer:^10^

□ a pop-up by every new NSAID prescription
□ a check list to assist in documenting these items

Please consider each rule by itself, as if it were the only rule being supported. Changes in the s*pecific things which should be documented* are allowed, assume that the computer will not recognize any other changes or exceptions.

On my computer, I would turn support: [i]

 □ **ON** □ **OFF**

[appears when ON is checked]**ON—because:**

 Please check all that apply:

□ I often don’t recognize that the conditions (new NSAID prescription) apply to the patient [i]
□ When the conditions apply, it is easy to forget to perform the action: [i]
  □ reviewing the risk factors
  □ recording the risk factors
  □ recording justification
□ Not following the rule (when it applies) can lead to serious harm to the patient^20^
  □ immediately
  □ after days
  □ after weeks
  □ after months
□ When the conditions apply, the action should be performed quickly^20^
□ This rule is relevant for performance indicators [i]
  □ which I find important
  □ which I am obligated to follow
□ I am responsible for performing this action [i]
  □ reviewing the risk factors
  □ recording the risk factors
  □ recording justification
□ Other reason:_______ [free text field]

[appears when OFF is checked] **OFF—because:**

Please check all that apply:

□ I do not want support, but it could be useful for: [i]
  □ full time doctors in my practice
  □ part time doctors in my practice
  □ new doctors in our practice
  □ supervisors
  □ junior/student doctors
  □ nurses
□ I will easily recognize when the conditions (new NSAID prescription) apply to the patient [i]
□ When the conditions apply, I won’t forget to perform the action [i]□ 
  □ reviewing the risk factors
  □ recording the risk factors
  □ recording justification
□ Decision support would interrupt my manner of working [i]
□ Decision support would be wrong too often^10,20^
□ Not following this rule would not lead to any serious harm for the patient^20^
□ The action does not need to be performed right away (it can be done later)^20^
□ There are no performance indicators relevant to this rule [i]
□ I am not responsible for performing this action [i]
  □ reviewing the risk factors
  □ recording the risk factors
  □ recording justification
□ Other reason:_______ [free text field]

     [i] indicates that a question resulted from interviews
